# An Attempt to Target Anxiety Sensitivity via Cognitive Bias Modification

**DOI:** 10.1371/journal.pone.0114578

**Published:** 2015-02-18

**Authors:** Elise M. Clerkin, Courtney Beard, Christopher R. Fisher, Casey A Schofield

**Affiliations:** 1 Department of Psychology, Miami University, Oxford, OH, United States of America; 2 Department of Psychiatry, Harvard University/McLean Hospital, Bellmont, MA, United States of America; 3 Department of Psychology, Skidmore College, Saratoga Springs, NY, United States of America; UNC Chapel Hill, UNITED STATES

## Abstract

Our goals in the present study were to test an adaptation of a Cognitive Bias Modification program to reduce anxiety sensitivity, and to evaluate the causal relationships between interpretation bias of physiological cues, anxiety sensitivity, and anxiety and avoidance associated with interoceptive exposures. Participants with elevated anxiety sensitivity who endorsed having a panic attack or limited symptom attack were randomly assigned to either an Interpretation Modification Program (IMP; *n* = 33) or a Control (*n* = 32) condition. During interpretation modification training (via the Word Sentence Association Paradigm), participants read short sentences describing ambiguous panic-relevant physiological and cognitive symptoms and were trained to endorse benign interpretations and reject threatening interpretations associated with these cues. Compared to the Control condition, IMP training successfully increased endorsements of benign interpretations and decreased endorsements of threatening interpretations at visit 2. Although self-reported anxiety sensitivity decreased from pre-selection to visit 1 and from visit 1 to visit 2, the reduction was not larger for the experimental versus control condition. Further, participants in IMP (vs. Control) training did not experience less anxiety and avoidance associated with interoceptive exposures. In fact, there was some evidence that those in the Control condition experienced less avoidance following training. Potential explanations for the null findings, including problems with the benign panic-relevant stimuli and limitations with the control condition, are discussed.

## Introduction

Anxiety sensitivity (AS) refers to the fear of anxiety-related physical sensations (e.g., racing heart) and cognitive symptoms (e.g., feeling “spaced out”) because of a belief that these symptoms have harmful consequences [1]. High levels of AS have been associated with the presence of anxiety disorders [2], [3], [4], depression [5], suicide [6] and difficulty exercising [7]. However, AS, or the “fear of fear,” has been most established as a core feature and risk factor for panic [8], [9], [10], [11].

Research has identified specific cognitive vulnerabilities underlying AS. Perhaps the most studied cognitive vulnerability is a negative interpretation bias for ambiguous AS-relevant experiences. For example, an individual with high AS might interpret their heart racing as indicative of a heart attack, while an individual with low AS might attribute this to walking up a flight of stairs or a natural physiological fluctuation. This tendency to make threatening interpretations of ambiguous physical and cognitive symptoms has been correlated with both high levels of AS [12], [13] and the presence of Panic Disorder (PD) [14] [15]. Interpretation bias is thus a target of existing psychotherapy treatments for PD, and decreases in interpretation bias related to bodily sensations predict subsequent symptom improvement during treatment for PD [16]. Moreover, prospective data suggest that interpretation bias is a risk factor for developing PD [13]. That said, relatively little is known about the relationship between interpretation bias and AS beyond these few correlational studies.

### Cognitive Bias Modification

Cognitive Bias Modification (CBM) procedures are designed to test the causal relationships between cognitive biases and emotional vulnerability. CBM tasks encourage changes in cognitive biases via repeated practice on tasks. Tasks are designed such that better performance implicitly facilitates the desired cognitive changes. Thus, explicit instruction to change the cognitive bias is not necessary, nor is explicit awareness of the purpose of the task. Such tasks have been used to establish causal relationships between a specific cognitive bias targeted and a wide range of emotional vulnerabilities [17], [18], [19]. Of note, the most recent meta-analysis evaluating the efficacy of these protocols across mood and anxiety disorders highlighted the efficacy of these interventions for promoting positive interpretation bias and decreasing negative mood state, but failed to find support for the reduction of emotional vulnerability in response to a stressor with these tasks [20]. Importantly, very little work has evaluated CBM as a potential intervention for anxiety sensitivity/panic disorder symptoms.

To our knowledge, two previous studies have attempted to test the causal relationship between interpretation bias, AS, and anxiety and avoidance associated with interoceptive exposures. Steinman and Teachman [21] developed a CBM task designed to induce a positive interpretation bias for AS-relevant information. Participants with elevated AS read AS-relevant scenarios and were asked to resolve a word fragment at the end of the scenario. As is typical of this type of scenario task [22], the word fragments always resolved the ambiguous scenario in a positive manner (in order to induce a positive interpretation bias). A single session of the positive CBM task successfully modified interpretations of new scenarios, as well as reduced AS symptoms on a self-report questionnaire, compared to two control conditions. There was also a small to moderate (but nonsignificant) effect in the hypothesized direction for individuals in the positive CBM group to report less fear after completing two interoceptive exposure tasks that were designed to elicit similar physiological sensations (Candle Blowing and Straw Breathing; see [21] for more information). Given that this effect did not reach statistical significance, and there was no statistically significant effect of condition on behavioral avoidance related to the interoceptive exposures, the authors highlight that further research evaluating the impact of CBM on emotional vulnerability will be important.

A more recent study evaluated a version of the CBM task, i.e., a modified Word-Sentence Association Paradigm (WSAP; [23], [24], [25], [26]) to test the impact of interpretation training on AS and emotional vulnerability [27]. In this study, the critical condition X time interaction was not significant; however, post-hoc analyses provided some evidence that CBM reduced AS and negative interpretations of physical symptoms compared to the sham condition. That said, similar to [21] the effect of condition on avoidance and self-reported fear during two interoceptive exposure tasks (Candle Blowing and Chair Spinning) was not significant, and the authors note that the results should be interpreted with caution given the study's small (*N* = 35) sample. Additionally, MacDonald and colleagues were unable to evaluate data from the WSAP Assessment (i.e., the manipulation check) because of the nonequivalence of the three versions of this task that were used. Further, given that authors interpreted post-hoc analyses following non-significant omnibus tests, replication is needed.

### Study Summary and Hypotheses

In an effort to replicate and extend prior work, the current study aimed to further test the causal relationship between interpretation bias and AS. As noted above, previous studies have been successful in modifying interpretation bias with subsequent effects on AS, but without significant effects on emotional vulnerability to a stressor [21], [27]. It is perhaps not surprising that a single session of a brief computer task was not powerful enough to reliably change behavior in high AS samples. In hopes of producing stronger effects, we increased the intervention dose from one to two visits. Like MacDonald and colleagues [27], we tested a modified Word-Sentence Association Paradigm [23], [24], [25], [26]. As noted by previous work [27], we speculated that this task would be a more powerful manipulation because it encourages individuals to both reject threatening interpretations and endorse benign interpretations compared to a scenario task that only encourages positive interpretations.

We hypothesized that individuals completing a “positive” training CBM task would endorse fewer threat interpretations and more benign interpretations of ambiguous panic-relevant situations than individuals completing a control task. We also expected the positive training group to report less AS after training than the control group. Finally, we did not expect the training to directly impact state anxiety. Rather, we expected changes in interpretation and AS to generalize to fear and behavioral responses only in response to interoceptive exposures that were designed to elicit a range of commonly reported physiological sensations experienced during panic.

## Method

### Participants

Participants (*N* = 65) were recruited from Miami University’s participant pool based on responses to the Anxiety Sensitivity Index (ASI) [28], [34] and one question regarding their experience of panic attacks. According to [29], individuals diagnosed with panic disorder with moderate or severe agoraphobia had a mean score of 32.1 (*SD* = 11.3) on the ASI. In the present study, participants who scored within ½ of a standard deviation of this clinical cutoff (> 26.45) and reported a panic attack or limited symptom attack were invited to participate. Specifically, participants were provided with a detailed description of panic attack and limited symptom attacks, taken from the *Panic Disorder Severity Scale-Self-report* (PDSS-SR) [30]. This resulted in a final sample of 65 participants (84.6% female; *M* Age = 18.59, *SD* = .77, *Range* 18–22). Race was reported as 1.5% American Indian/Alaskan Native; 1.5% Asian; 1.5% Black/African American, 93.8% White, and 1.5% Bi- or multiracial. Ethnicity was reported as 100% Not Hispanic/Latino.

The experiment consisted of two visits that were separated by two days for most participants (*M* = 2.44; *Median* = 2.00 days); length of time between the two sessions did not significantly differ between conditions (*t*_(63)_ = .81, *p* = .42, d = .20). Only participants who completed both sessions of training were included in analyses (*N* = 3 non-completers).

### Materials

**Baseline panic symptoms, mood, and anxiety.** The 7-item *Panic Disorder Severity Scale-Self-report* (PDSS-SR) [30] was included to characterize the sample, and to evaluate the frequency, distress, and impairment associated with panic attacks (α = .84). The *Patient Health Questionnaire-4* (PHQ-4) [31], a 4-item scale, was included to assess baseline symptoms of anxiety and depression (α = .75). Finally, the 6-item *Brief State Anxiety Measure* (BSAM) [32] is composed of items from the State-Trait Anxiety Inventory [33]; higher numbers reflect more anxiety. The BSAM was used to evaluate the impact of training on symptoms of state anxiety (average α = .86).

**Interpretation Modification for Panic vs. Control training.** The *Interpretation Modification Program-Panic* (IMP) (i.e., Positive Training condition) is a modified version of the Word-Sentence Association Paradigm (WSAP) that repeatedly reinforces participants for making benign interpretations of ambiguous situations. Given that the WSAP used in previous research targets fears specific to social anxiety, panic-relevant stimuli were developed for IMP used in the current study. When developing IMP, five independent judges with a doctorate in clinical psychology rated the extent to which threat and benign words were panic-relevant on a 0–4 scale, anchored at 0 (Not at all panic-relevant) and 4 (Extremely panic-relevant). Words for which the mean panic-relevance rating was less than two were removed. Mean panic relevance for threat words was 3.23 and 0.42 for non-threat words. These same five judges rated the extent to which words in the Control training were related to the corresponding sentences. The rating scale was anchored at 0 (“Not at all related”) and 4 (“Extremely related”). The mean rating was 3.31 for related words and 0.09 for unrelated words.

On a given trial, a panic-relevant threat or non-threat word appeared for 500 ms, followed by an ambiguously related sentence. Participants were asked to indicate whether the word and sentence were related. The program guided interpretation by providing feedback (“You are correct!”) following the acceptance of a benign interpretation (e.g., exercise) as related to a sentence (e.g., “After climbing the stairs your heart begins to beat harder than usual”), and negative feedback (“You are incorrect”) following the acceptance of a threatening interpretation (e.g., heart attack) as related to the same sentence. Thus, participants in the IMP condition were positively reinforced for benign interpretations and corrected for negative/threat interpretations. See Table 1 for additional examples.

**Table 1:** Example WSAP sentences paired with threatening and benign associations.

Choking (Threat)	You wake up in the morning and it is hard to swallow
Thirsty (Benign)	You wake up in the morning and it is hard to swallow
Heart Attack (Threat)	After climbing the stairs your heart begins to beat harder than usual
Exercise (Benign)	After climbing the stairs your heart begins to beat harder than usual
Embarrassing (Threat)	You feel like you might faint in line at the store and people would be around to see
Helpful (Benign)	You feel like you might faint in line at the store and people would be around to see

In the *Control* condition, the computer presented words that were not related to the panic-relevant interpretation of the sentence, but were related or unrelated to the sentence itself (e.g., “staircase” vs. “table”–“After climbing the stairs your heart begins to beat harder than usual”). Participants in the Control group saw the identical ambiguous situations displayed in the IMP condition, and they received positive or negative feedback about the accuracy of their responses. Thus, Control training was equated for the effects of time, feedback, computer usage, exposure to ambiguous scenarios, and attention from the researcher [25]. Across both conditions, threat/non-threat (IMP) or related/unrelated (Control) words were paired with 65 sentences, resulting in a total of 130 trials for each condition. One session of training lasted approximately 10 minutes.

**Evaluation of training: Interpretation bias and anxiety sensitivity.** To assess interpretation biases an assessment version of the *Word-sentence Association Paradigm* (WSAP-A) was used. The WSAP-A is identical to IMP training (no new word-sentence associations were included), except that the training contingencies are removed in the WSAP assessment. In other words, participants did not receive feedback when they determined whether the threatening and benign interpretations were related or unrelated to the ambiguous sentences. This task was designed to measure changes in interpretation biases as a result of IMP (vs. Control) training. The current study evaluated two potential bias scores: % endorsement of threat and % endorsement of benign words, following [24].

The *Anxiety Sensitivity Index* (ASI) [28], [34] is a 16-item scale with adequate psychometric properties that assesses an individual’s concerns about the negative consequences associated with anxiety (e.g., ‘‘It scares me when my heart beats rapidly”). It was used to recruit participants, and to evaluate changes in anxiety sensitivity as a function of training. Internal consistency was acceptable at all time points, although it was lower for preselection (vs. within-session) scores (Preselection α = .67; Visit 1 α = .84; Visit 2 α = .82).

**Evaluation of training: Emotional vulnerability.** During the post-training assessment, participants were asked to complete three randomly ordered interoceptive exposure tasks (Task 1: Jumping Jacks, where participants were asked to engage in callisthenic jumps; Task 2: Candle Blowing, where participants were asked to pretend to forcefully blow out a candle; Task 3: Chair Spinning, where participants were asked to rapidly spin in their chairs). These tasks induce a range of benign, temporary physiological sensations commonly experienced during panic (e.g., racing heart, shortness of breath, dizziness), and are adapted from tasks used in cognitive behavioral treatments for panic [35]. For each of these tasks, participants were asked to engage in the activity for up to 2 minutes if they were willing. They were told that they could stop at any time before 2 minutes passed. In order to assess participants’ peak anxiety in response to panic-relevant physical sensations, participants completed a BSAM rating prior to the IMP task and immediately following each interoceptive exposure task. Participants also reported which of the three tasks was most anxiety producing.

### Procedure

As part of the consent process, participants were told that the purpose of the study was to evaluate how individuals perceive the relationship between words and sentences, and how these word-sentence associations may be related to the experience of harmless physiological sensations. Following informed consent, participants were assigned to IMP (*n* = 33) or Control (*n* = 32) training according to a randomized block design. Participants and experimenters were both blind to condition. During Visit 1, participants first completed demographic and descriptive information, followed by baseline symptoms of panic, anxiety, and depression (PDSS-SR, PHQ-4). Next, participants completed the baseline measures of interpretation bias (WSAP-A) and anxiety sensitivity (ASI). Finally, participants completed either the IMP or Control training. Immediately prior to and following training, the BSAM was administered to check for direct effects of training on state anxiety.

During Visit 2, participants first completed either the IMP or Control training. Again, the BSAM was administered immediately prior to and following training. Next, post-training measures were collected to evaluate the impact of training on interpretation bias (WSAP-A) and anxiety sensitivity (ASI). Finally, to assess whether training influenced emotional vulnerability, participants were guided through the three interoceptive exposure tasks, which were administered in a random order. As well, participants engaged in a brief exit questionnaire to determine whether they recognized the pattern of how the computer directed them to respond (response choices: 1) Yes to mostly negative word sentence associations; 2) Yes to mostly positive word sentence associations; 3) No to mostly negative word sentence associations; 4) No to mostly positive word sentence associations; 5) there was not a pattern).

### Ethics Statement

All participants in the present study were 18+ years old. All participants provided their written consent to participate in this study, and research procedures were approved by the Institutional Review Board at Miami University (approval # 00225i).

## Results

### Sample Characteristics

Table 2 shows sample characteristics by training condition. Chi-square tests indicated no significant group differences for gender (χ^2^_(1, *N* = 65)_ = .55, *p* = .46) or race (coded as White vs. Other; χ^2^_(1, *N* = 65)_ = .001, *p* = .98). T-tests indicated that there were no statistically significant differences between the training conditions for age (*t*_(62)_ = .97, *p* = .34, *d* = .24), panic symptoms assessed with the PDSS-SR (*t*_(63)_ = .97, *p* = .34, *d* = .24), or baseline depression and anxiety symptoms assessed with the PHQ-4 (*t*_(63)_ = .49, *p* = .63, *d* = .12). Please see the attached data file S1 Data.

**Table 2:** Baseline and preselection sample characteristics.

	Training Condition					
	IMP		Control		Full Sample	
	*M* or *N*	*SD* or *%*	*M* or *N*	*SD* or *%*	*M* or *N*	*SD* or *%*
Age	18.50	.672	18.69	.859	18.59	.77
Gender (Female)	29	87.9%	26	81.3%	55	84.6%
Race						
Alaskan Native/American Indian	0	0.0%	1	3.13	1	1.5%
Asian	1	3.0%	0.0%	0.0%	1	1.5%
Black	1	3.0%	0.0%	0.0%	1	1.5%
White	31	93.9%	30	93.8%	61.00	93.8%
Bi- or multiracial	0	0.0%	1	3.13	1	1.5%
ASI (preselection)	36.64	7.56	34.75	7.47	35.71	7.52
PDSS-SR	4.76	3.48	5.72	4.45	5.24	3.99
PHQ-4	3.90	2.31	3.63	2.38	3.77	2.33

*Note*: IMP is the *Interpretation Modification* Program, *ASI* (preselection) is the *Anxiety Sensitivity Index*, PDSS-SR is the *Panic Disorder Severity Scale-Self-report*, *and* PHQ-4 is the *Patient Health Questionnaire-4*.

### Validity Check: WSAP-A

We conducted post-hoc correlations to determine whether Threat Endorsement and Benign Endorsement were correlated with anxiety sensitivity, as assessed with the ASI. Supporting the validity of the Threat Endorsement variable, greater Threat Endorsement was significantly related to greater self-reported anxiety sensitivity at both Visit 1 (*r* = .43, *p* = .001) and Visit 2 (*r* = .41, *p* = .001). However, the Benign Endorsement variable was not significantly associated with self-reported anxiety sensitivity at either Visit 1 (*r* = -.05, *p* = .68) or Visit 2 (*r* = -.20, *p* = .12).

### Training Condition Manipulation Check

**Fig 1:**
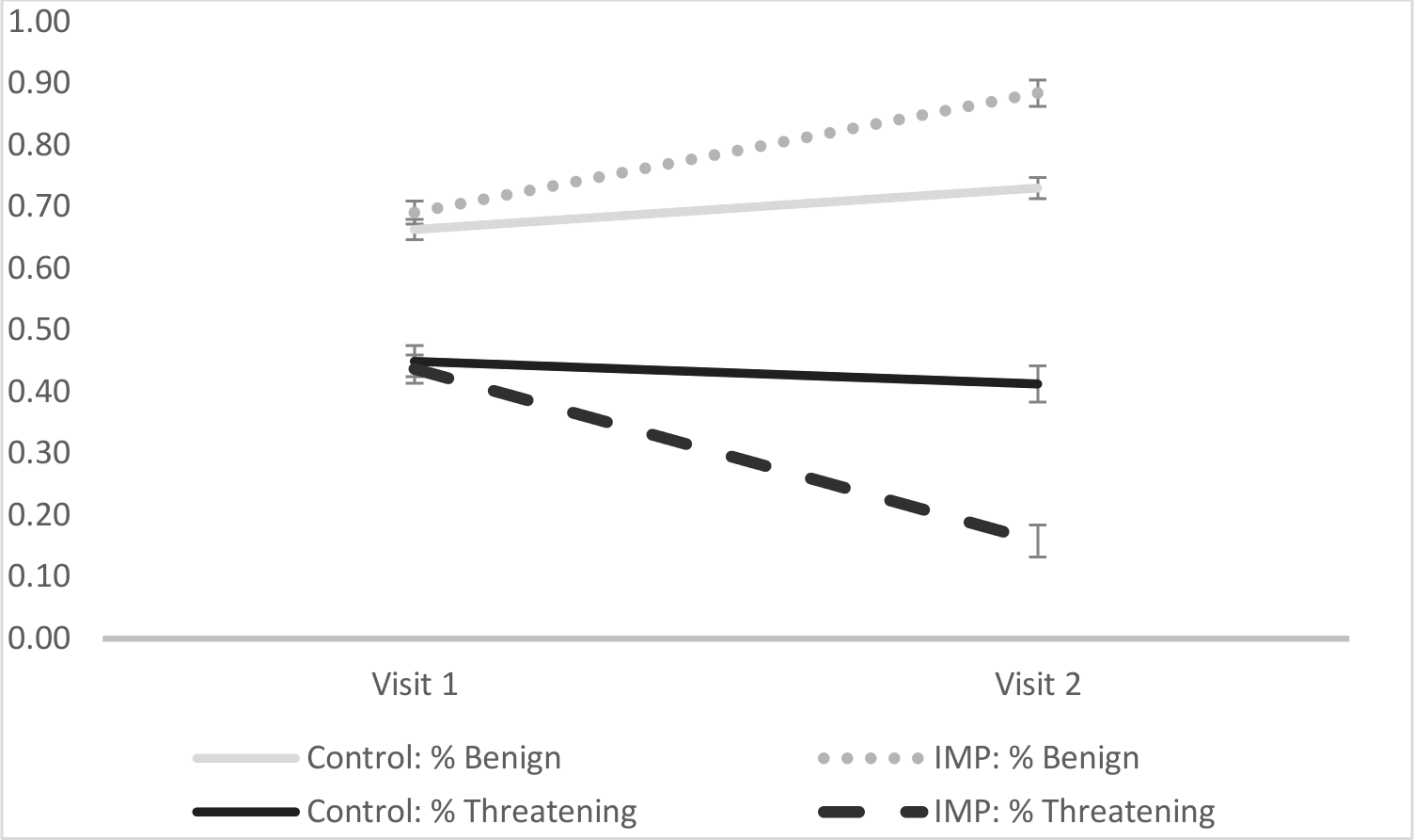
Change in WSAP as a function of Condition.

To evaluate the influence of condition on the endorsement of threatening vs. benign interpretations (WSAP-A), we conducted a 2 (Training Condition: IMP vs. Control) X 2 (Time: Visit 1 vs. Visit 2) X 2 (Valence: % Threatening vs. % Benign interpretation) repeated measures ANOVA, with training condition as the between-subjects variable, and time and valence as the within-subjects variables. In line with expectations, there was a significant training condition by time by valence interaction (*F*_(1, 61)_ = 58.96, *p* < .001, η^2^_p_ = .49). Importantly, a follow-up repeated measures ANOVA conducted within each condition separately showed that there was no significant effect of Time within the Control group (*F*_(1, 30)_ = 1.24, *p* = .27, η^2^_p_ = .04), but there was a significant effect of Time within the IMP group (*F*_(1, 31)_ = 8.65, *p* = .006, η^2^_p_ = .22). Follow-up t-tests indicated that there was not a significant between-groups difference for percentage of threatening or benign interpretations endorsed at Visit 1 (% Threatening: *t*_(62)_ = .38, *p* = .71, *d* = .09; % Benign: *t*_(62)_ = 1.10, *p* = .27, *d* = .28). As expected, participants in the IMP (vs. Control) condition endorsed fewer threatening (*t*_(62)_ = 6.50, *p* < .001, *d* = 1.62) and more benign (*t*_(62)_ = 5.64, *p* < .001, *d* = 1.41) interpretations at Visit 2. See Fig. 1.

Within-subjects tests indicated that within the IMP condition, participants were significantly more likely to endorse benign words (IMP: *t*_(31)_ = 10.12, *p* < .001, d = 1.79) and reject threatening words (IMP: *t*_(31)_ = 9.21, *p* < .001, d = 1.63) at visit 2 vs. visit 1. Within the Control condition, participants were also significantly more likely to endorse benign words at visit 2 vs. visit 1 (*t*_(30)_ = 4.07, *p* < .001, d = .73); however, within the Control group, there was no statistically significant difference in the rejection of threatening words at visit 2 (vs. visit 1; Control: *t*_(30)_ = 1.80, *p* = .08, d = .32). In sum, both threatening and benign interpretations changed in the anticipated direction for the IMP group, suggesting that training was effective in manipulating interpretation bias. Interestingly, findings also suggest that all participants, regardless of training condition, learned to endorse more benign words at visit 2 vs. visit 1 (although the effect size for this change was more than twice as large in the IMP group compared to the control).

Finally, to evaluate whether training directly influenced state anxiety (assessed with the BSAM), we conducted a 2 (Condition: IMP vs. Control) X 2 (Time: Pre- vs. Post-training) repeated measures ANOVA. Consistent with expectations, there was not a significant Condition by Time interaction at either time point (Visit 1: *F*_(1, 63)_ = 3.21, *p* = .08, η^2^_p_ = .05; Visit 2: *F*_(1, 63)_ = .26, *p* = .61, η^2^_p_ = .004).

### Effects of Training Condition on Change in Anxiety Sensitivity

**Fig 2:**
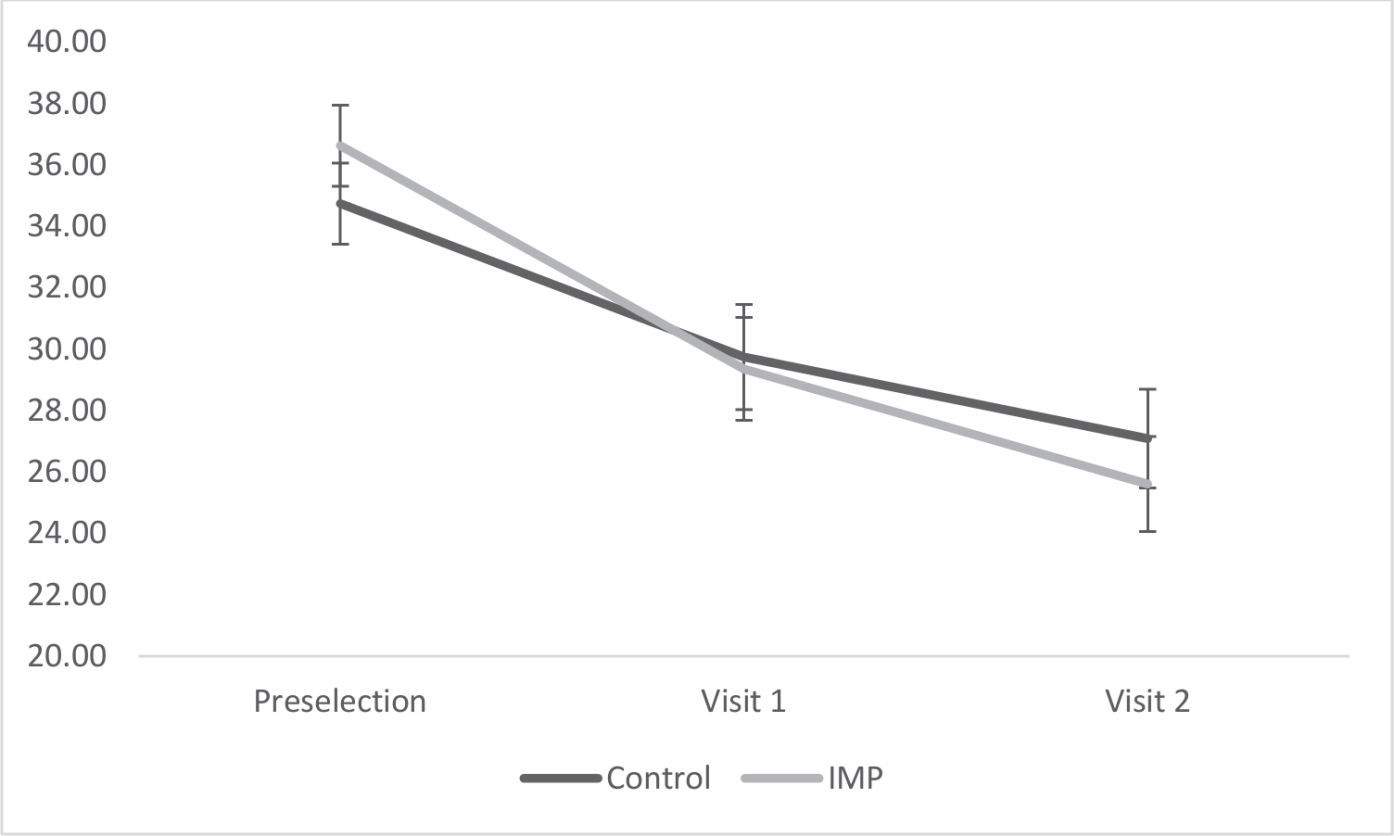
Change in Anxiety Sensitivity as a function of Condition.

To evaluate the influence of condition on self-reported anxiety sensitivity, assessed with the ASI, we conducted a 2 (Training Condition: IMP vs. Control) X 3 (Time: Preselection vs. Visit 1 vs. Visit 2) repeated measures ANOVA, with training condition as the between-subjects variable, and time as the within-subjects variable. Contrary to expectations, there was not a significant training condition by time interaction (*F*_(2, 126)_ = 1.49, *p* = .23, η^2^_p_ = .02), and there was a significant main effect for time (*F*_(2, 126)_ = 45.36, *p* < .001, η^2^_p_ = .42). Across both conditions, ASI scores reduced from preselection to Visit 1, and from Visit 1 to Visit 2 (all *p* < .05). See Fig. 2.

### Effects of Training Condition on Emotional Vulnerability

To evaluate the influence of condition on emotional vulnerability to a physiological stressor, we first conducted a multivariate ANOVA with one between-subjects variable (Training Condition: IMP vs. Control) and three outcomes (Peak Anxiety, assessed with the BSAM, for Jumping Jacks, Candle Blowing, and Chair Spinning). There was a significant main effect for Condition (*F*_(1, 60)_ = 3.52, *p* = .02, η^2^_p_ = .15), but tests of between-subjects effects indicated that there were no significant group differences for any of the three outcomes as assessed independently (Jumping Jacks: [*F*_(1, 62)_ = 3.57, *p* = .06, η^2^_p_ = .05, Control *M* = 15.28, *SD* = 4.19; IMP *M* = 17.19, *ST* = 3.87]; Candle Blowing: [*F*_(1, 62)_ = 2.55, *p* = .12, η^2^_p_ = .04, Control *M* = 13.91, *SD* = 4.06, IMP *M* = 15.56, 4.23]; Chair Spinning: [*F*_(1, 62)_ = .97, *p* = .33, η^2^_p_ = .02, Control *M* = 17.81, *SD* = 4.22, IMP *M* = 16.75, *SD* = 4]). Further, an independent samples t-test indicated that there was no significant group difference when evaluating peak anxiety for the task participants rated as most anxiety provoking (*t*_(63)_ = .89, *p* = .38, *d* = .22, *Control M* = 17.84, *SD* = 4.06, IMP *M* = 18.73, *SD* = 3.96).

Finally, we conducted a multivariate ANOVA with one between-subjects variable (Training Condition: IMP vs. Control) and three outcomes (Time spent on Jumping Jacks, Candle Blowing, and Chair Spinning). Contrary to expectations, there was no significant impact of Condition on Time (*F*_(3, 60)_ = 1.50, *p* = .22, η^2^_p_ = .07). There was a statistically significant effect of Condition on Time associated with the physical sensation task rated as most anxiety provoking (*t*_(63)_ = 2.17, *p* = .03, *d* = .54). Surprisingly, participants in the Control (*M* = 113.49, *SD* = 18.06) condition engaged in the task for longer, as compared to those in the IMP condition (*M* = 100.84, *SD* = 27.85).

### Participant Awareness of Training Pattern

There was no significant group difference across condition in terms of participants’ perception of the pattern in which the computer directed them to respond (χ^2^_(1, *N* = 65)_ = 3.82, *p* = .43). Most participants believed that the computer directed them to respond with “Yes” to mostly positive word-sentence associations (IMP *n* = 19, 57.6%; Control *n* = 18, 56.3%).

## Discussion

The current study goals were to investigate an adaptation of a Cognitive Bias Modification procedure designed to influence interpretations related to anxiety sensitivity, and to evaluate the causal relationships among interpretation bias, anxiety sensitivity, and anxiety and avoidance associated with a series of interoceptive exposures. This study advances prior research efforts by providing a test of multiple sessions of a Word-Sentence Association Paradigm in a sample with elevated anxiety sensitivity in an effort to strengthen observed training effects. Further, we included three interoceptive exposures to elicit a range of commonly reported physiological sensations experienced during panic, as opposed to including just two interoceptive exposures designed to elicit similar sensations.

As predicted, we found that individuals in the IMP (vs. Control) training condition had healthier interpretations as evidenced by fewer threatening and more benign word-sentence associations following training. However, contrary to expectations, there was not a significant impact of training on self-reported anxiety sensitivity (assessed with the ASI), or most measures of anxiety and avoidance associated with interoceptive exposure exercises. Surprisingly, when compared to those in the Control condition, individuals in the IMP condition demonstrated greater avoidance associated with the interoceptive exposure exercise rated as most anxiety provoking.

While this study was helpful from an initial “proof of concept” standpoint to demonstrate that AS-relevant interpretations could be experimentally manipulated via the modified WSAP, the lack of significant group differences in most other outcomes, along with the greater avoidance among the IMP condition, were unexpected. There are a few possible explanations for these findings. First, changing interpretation bias may not be an effective way to influence AS. This seems quite unlikely given that biased interpretation of anxiety-relevant symptoms is the hallmark of AS, changes in interpretation bias predict changes in panic symptoms, and previous research suggests that AS may change with a CBM targeting interpretation [21],[27]. A more credible explanation is that the CBM task did not induce robust changes in interpretation. A limitation of this study is that we did not include an independent measure of interpretation bias; thus, we do not know if interpretation changes evident on the WSAP would have generalized to other measures of interpretation. As well, future research should include new word-sentence associations (not previously seen during training) in the assessment of interpretation bias.

It is also possible that the control condition unintentionally affected AS. Indeed, individuals across both conditions improved in anxiety sensitivity (assessed with the ASI) and benign interpretations (assessed with the WSAP-A) across time points. Further, contrary to expectations, those in the Control (vs. IMP) condition exhibited less avoidance by engaging in the physical sensation task rated as most anxiety provoking. From an associative learning perspective, individuals in the Control condition were still repeatedly determining whether words were related or unrelated to panic-relevant scenarios, which could have strengthened associations between panic-relevant information and benign/neutral information. Future research using alternate control conditions (e.g., a control condition that does not include any panic relevant words or sentences) is needed.

Notwithstanding this possible explanation, given that participants in both conditions demonstrated reductions in self-reported AS from pre-selection to Visit 1, it is also possible that scores were simply regressing to the mean [36]. To test for this, future researchers may consider incorporating a multiple baseline design as an additional control that will allow them to isolate and control for spontaneous improvement over time. Including a multiple baseline design in conjunction with a typical Control group would also help us better understand the reasons why individuals in the Control condition sometimes improve across CBM trials [37]. Namely, such a design could determine whether improvement is due to nonspecific improvement and regression to the mean prior to receiving any CBM task versus whether improvement is due to something about the Control training. Either way, this research points to a valuable, often neglected consideration for CBM researchers: that baseline scores and Control groups should be carefully considered when evaluating change processes.

Finally, the IMP task may not have induced the desired changes in part due to the stimuli. Although both Threat and Benign Endorsement changed as anticipated within the IMP condition, the post-hoc validity check evaluating the relationships between Threat and Benign Endorsement and self-reported anxiety sensitivity suggest that Threat Endorsement was a more valid indicator of anxiety sensitivity than Benign Endorsement. In particular, while Threat Endorsement was significantly related to self-reported AS at both time points, Benign Endorsement was not significantly related to self-reported AS at either time point. This is interesting in light of previous research that training participants to endorse positive interpretations to ambiguous scenarios resulted in decreases in anxiety sensitivity [21]. In contrast, the current study tested a different CBM task, i.e., the modified Word-Sentence Association Paradigm, because of its potential to not only train participants to endorse benign interpretations, but also to reject threatening interpretations. Given this, it may be meaningful that in the present study, the measure tied to rejecting threatening (vs. endorsing benign) interpretations was more strongly related to AS. It is possible that to modify AS, the active generation of more benign AS-relevant interpretations is more instrumental than the rejection or endorsement of threatening AS-relevant interpretations. If this is true, future research will be needed to ensure that the Benign Endorsement portion of the WSAP is equally as valid as the Threat Endorsement portion. As well, further piloting of stimuli may ensure that the benign and threatening stimuli are both logically related to the sentences. Anecdotally, although our pilot data supported the use of these stimuli, we believe there is much room for improvement in the benign words associated with the ambiguous sentences.

In sum, this project provides a replication of previous research [27] indicating that it is possible to directly modify panic-relevant interpretations in a high AS sample using an interpretation modification paradigm (IMP; modified Word-Sentence Association Paradigm). However, changes on the WSAP-A did not affect AS or emotional vulnerability as anticipated. These findings are consistent with a recent meta-analysis evaluating the efficacy of CBM tasks targeting interpretation biases that suggested that emotional vulnerability is not attenuated by these tasks across studies of mood and anxiety symptoms [20]. Based on previous work and the findings of this study, improvements to the study's AS-relevant stimuli, specifically the benign words, may be needed in order to comprehensively understand the potential efficacy of CBM for AS symptoms. As well, the stimuli and task will need to be evaluated within a more diverse sample given that our population was largely White, young, and female. Should an improved task and protocol be developed, the WSAP could potentially yield a transdiagnostic tool for the many individuals whose elevated anxiety sensitivity causes significant impairment or distress; however, more work is needed in this area before firm conclusions can be drawn about the clinical usefulness of CBM in influencing interpretations and AS.

## Supporting Information

S1 Data Data file. For additional information please contact clerkiem@miamiOH.edu.
(XLS)
